# Protocol for the development of a core outcome set for pelvic girdle pain, including methods for measuring the outcomes: the PGP-COS study

**DOI:** 10.1186/s12874-018-0624-5

**Published:** 2018-12-03

**Authors:** Francesca Wuytack, Annelie Gutke, Britt Stuge, Siv Mørkved, Christina Olsson, Hilde Stendal Robinson, Nina K. Vøllestad, Birgitta Öberg, Lena Nilsson Wikmar, Juan Jose Saldaña Mena, Valerie Smith

**Affiliations:** 1 School of Nursing & Midwifery, Trinity College Dublin, 24 D’Olier Street, Dublin 2, Ireland; 2 Department of Health and Rehabilitation, Institute of Neuroscience and Physiology, University of Gothenburg, Box 455, 40530 Göteborg, Sweden; 3 Division for Neuroscience and Musculoskeletal Medicine, Oslo University Hospital, Oslo, Norway; 4 Department of Public Health and General Practice, Norwegian University of Science and Technology, Medisinsk teknisk forskningssenter, N-7489 Trondheim, Norway; 5 Mörby Academic Primary Healthcare Centre, Golfvägen 8, 182 31 Danderyd, Sweden; 6 Department of Health Sciences, Institute of Health and Society, University of Oslo, Postboks 1089 Blindern, 0318 Oslo, Norway; 7 Institute of Health and Society, University of Oslo, Box 1133 Blindern, N-0318 Oslo, Norway; 8 Department of Medicine and Health, Linköping University, 581 85 Linköping, Sweden; 9 Department of Neurobiology, Care Sciences and Society, Division of Physiotherapy, Karolinska Institutet, Stockholm, Sweden; 10 Universidad Estatal del Valle de Ecatepec, Calle León Guzmán #60 Col Valle de Anáhuac CP, 55210 Ecatepec de Morelos, Mexico

**Keywords:** Pelvic girdle pain, Core outcome set, Delphi survey, Outcome measurement, Consensus

## Abstract

**Background:**

Pelvic Girdle Pain (PGP) is an important cause of disability and economic cost worldwide. There is a need for effective preventative and management strategies. Emerging studies measure a variety of outcomes rendering synthesis and translation to clinical practice difficult. A Core Outcome Set (COS) can address this problem by ensuring that data are relevant, useful and usable for making well-informed healthcare choices. The aim of this study is to develop a consensus-based PGP-COS, including agreement on methods (e.g. instruments) for measuring the construct outcomes in the COS for use in research and clinical practice. Furthermore, as there is uncertainty as to whether incorporating stakeholder interviews in addition to conducting a systematic review to determine an initial list of outcomes for the Delphi survey, or, whether using different rating scales in a Delphi survey impacts on the final COS, we propose to embed two methodological studies within the PGP-COS development process to address these questions.

**Methods:**

The PGP-COS study will include five phases: (1) A systematic review of the literature and semi-structured interviews with 15 patients (three countries) to form the initial list of outcomes for the Delphi survey; (2) A 3-round Delphi including patients, clinicians, researchers and service providers; (3) A systematic review of methods for measuring the outcomes in the preliminary PGP-COS identified in the Delphi survey; (4) A face-to-face consensus meeting to agree on the final PGP-COS and methods for measuring the COS; (5) Global dissemination.

To address the methodological questions, we will assess the number and type of outcomes, in the final PGP-COS, that were exclusively derived from the interviews. Secondly, we will randomise Delphi survey participants to either a 5-point or 9-point importance rating scale, and examine potential differences in ‘important’ ratings between the groups.

**Discussion:**

There is currently no COS for measuring/monitoring PGP in trials and clinical practice. A PGP-COS will ensure that relevant outcomes are measured using appropriate measurement instruments for patients with PGP globally.

**Core outcome set registration:**

This PGP-COS was registered with COMET (Core Outcome Measures for Effectiveness Trials) in January 2017 (http://www.comet-initiative.org/studies/details/958).

**Electronic supplementary material:**

The online version of this article (10.1186/s12874-018-0624-5) contains supplementary material, which is available to authorized users.

## Background

Low back pain is a leading cause of Years Lived with Disability (YLD) [1], is the sixth largest contributor to Disability Adjusted Life Years (DALYs) [2] and is associated with high costs of healthcare utilisation and absenteeism from work. Pelvic Girdle Pain (PGP) has sometimes been considered one of the main subgroups of low back pain; however, currently experts agree that PGP has distinct symptomatology and that differentiation between low back pain from lumbar origin and PGP is essential for appropriate management [3]. Moreover, PGP may include symphysis pubis pain, which cannot be classified as low back pain. Subsequently, PGP is a distinct condition, and treatment, examination and outcomes should be studied taking this into account to increase our understanding of PGP.

Pelvic Girdle Pain can be related to blunt or repetitive trauma, osteoarthritis or arthritis, and can as such affect both men and women. In most cases there is no clear explanation for the onset of PGP, but it is particularly common during pregnancy and most studies have been conducted on pregnant or postpartum women. Very little is known about other PGP groups due to a lack of studies. Pelvic Girdle Pain affects around half to two thirds of women at some point during pregnancy [4, 5] and 8.5% continue to have significant symptoms two years postpartum [6]. It is also the number one cause of sick leave during pregnancy [7–9] with large economic, social and psychological impact on individual families and the wider society. There is an urgent need for effective preventative and management strategies to address this personal and societal burden for women in fertile age [3, 10]. However, emerging studies measure a variety of outcomes and measure the same outcomes by different methods [5, 10], rendering synthesis and translation to clinical practice difficult and sometimes impossible.

A core outcome set (COS) is a standardised set of outcomes which should be measured and reported worldwide, as a minimum, in all studies for a specific health area/condition [11], and many COS across different healthcare fields have been developed, for example, COS in non-specific low-back pain complex regional pain syndrome [12, 13]. A COS allows for findings to be combined, compared and contrasted, reduces potential for reporting bias, and ensures that the data are useful and usable, which is essential for making well-informed healthcare choices. There was no registered or published COS for PGP. We registered this PGP-COS with COMET (Core Outcome Measures for Effectiveness Trials) in January 2017 (http://www.comet-initiative.org/studies/details/958). COMET is an initiative that focuses on the development and application of COS. They aim to collate relevant resources in the COMET database, have published the COMET handbook on developing COS and provide an online registry for COS projects to promote transparent COS development (see http://www.comet-initiative.org/).

## Methods/design

### Aim

To describe the protocol to develop a consensus-based PGP-COS for use in research and clinical practice so as to promote the health and well-being of women with PGP globally, by ensuring consistent and relevant outcome measurement and reporting in any prevention and management strategy.

## Objectives

1. To collate a list of reported outcomes from a systematic review of the literature of studies on PGP and from patient interviews for use in a Delphi survey;
2. To develop consensus on a preliminary PGP-COS, using the list of outcomes from Objective 1, via a 3-round international electronic Delphi survey;
3. To synthesise the evidence on the available outcome measurement instruments for measuring the construct outcomes (e.g. pain) in the preliminary PGP-COS;
4. Using the preliminary PGP-COS (Objective 2), develop a final PGP-COS for use in all future studies on PGP, and in clinical practice, including how to measure the outcomes in the COS, via an international face-to-face consensus meeting with key stakeholders;
5. To disseminate and promote the implementation of the COS to key stakeholders internationally;

In addition, we have two methodological objectives:

6. To determine if including patient interviews in addition to a systematic review to identify the initial list of outcomes influences the final COS;
7. To evaluate any differences in using a 9-point versus a 5-point rating scale in the Delphi survey, in terms of its impact on the number of ‘important’ ratings received for each outcome in each round of the survey and on the final PGP-COS.

### Design

**Fig. 1:**
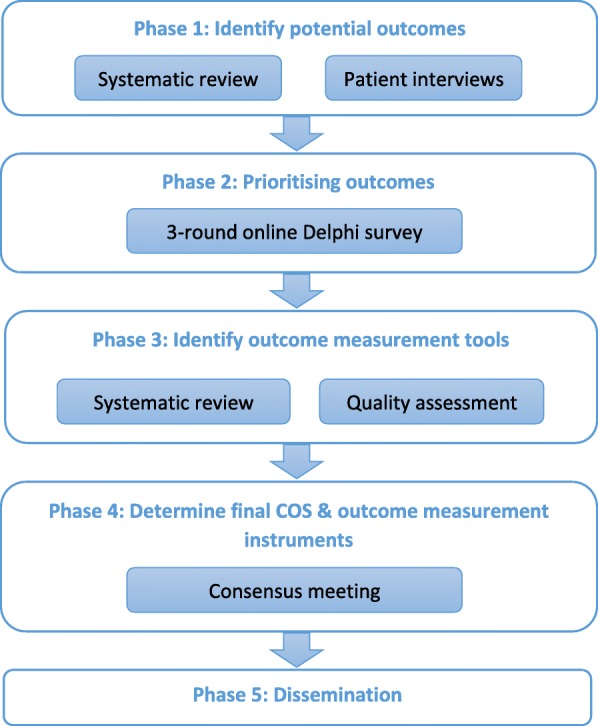
Overview of the PGP-COS development study

A consensus Delphi survey and face-to-face meeting will be used to identify and agree on a PGP-COS, informed by comprehensive systematic reviews of outcomes and outcomes measurement instruments for PGP. Figure 1 provides an overview of the study phases. The PGP-COS project plan was registered with COMET and is publically available to avoid duplication (http://www.comet-initiative.org/studies/details/958). This protocol was developed in accordance with the COMET handbook [14] and COSMIN (Consensus-based Standards for the selection of health Measurement Instruments) guidance [15]. The study steering committee will include researchers, clinicians, methodologists and at least one patient representative, and will meet quarterly to discuss progress and monitor the conduct of the study. In case of any significant changes to this protocol in future, these will be communicated to the ethics committee, the journal and the funders.

### Scope

This PGP-COS and corresponding outcome measurement instruments is aimed at monitoring the progress of PGP in both research (randomised and non-randomised studies) and clinical practice. The target population is women with PGP during pregnancy or postpartum (defined as any time after a birth), as this is by far the most common presentation. Very little is known about other, smaller groups with PGP due to a lack of research; subsequently this COS focuses on PGP in pregnant and postpartum women. We hope that as further literature emerges concerning other PGP groups, we will be able to expand or adapt this COS for these groups in future studies, where appropriate. PGP is defined as pain between the posterior iliac crest and the inferior gluteal fold, particularly in the vicinity of the sacroiliac joints, that may radiate in the posterior thigh and can occur in conjunction with or separately in the symphysis pubis [3]. However, we will exclude pain of the pelvic girdle attributable to specific pathologies such as inflammatory arthropathies, infection, and trauma.

### Phase one: Generating the list of potential outcomes

#### Systematic search of the literature

We will conduct a systematic review of the literature to identify outcomes measured in intervention studies and systematic reviews related to PGP. We chose to limit the review to these study types as our PGP-COS will be developed for effectiveness trials, and follows COMET’s mission. We will search the following databases from inception: PubMed, the Cochrane Library, PEDro and Embase. Two reviewers will independently select relevant records by title/abstract and full-text, using the selection criteria outlined in Table 1. Disagreement will be resolved through discussion or if necessary by involving a third reviewer. Health, clinical, social and economic outcomes (and their verbatim definitions) will be extracted and their corresponding outcome measurement instruments, where reported, will be recorded. The quality of outcome reporting will be assessed using the six questions proposed by Harman et al. [16]. Subsequently we will group the outcomes into outcome domains using the OMERACT Filter 2.0 framework [17], but all identified outcomes will be presented for rating in the Delphi survey.

**Table 1:** Selection criteria for the systematic review of outcomes used in the existing PGP literature

Population	Women with PGP during or after pregnancy. PGP is defined as pain between the posterior iliac crest and the inferior gluteal fold, particularly in the vicinity of the sacroiliac joints, that may radiate in the posterior thigh and can occur in conjunction with or separately in the symphysis pubis [3]. We will exclude studies that include women with PGP and low back pain without differentiation between the two. We will also exclude studies that examine a population with PGP resulting from specific pathologies (e.g. infection, spondyloarthropaties, trauma).
Intervention	Any intervention aimed to treat/prevent PGP
Comparator	Any comparator intervention or control
Outcome	Any outcome measured to assess/monitor PGP
Study design	Intervention studies (randomised or non-randomised), systematic reviews of interventions

#### Interviews with patients

We will conduct semi-structured interviews using open-ended questions with a purposive sample of approximately 15 pregnant/postpartum women with PGP (5 in Ireland, 5 in Sweden, 5 in Mexico), because those experiencing PGP directly, might indicate different outcomes of importance than those measured by clinicians/researchers in studies [18]. Participants must be aged 18 years or more and have PGP (as primary complaint) at the time of the interview. PGP is defined as pain between the posterior iliac crest and the inferior gluteal fold and/or at the symphysis pubis [3], and confirmed by a qualified clinician following a physical examination. A pragmatic approach will be used in that the clinician will identify patients with PGP at his/her discretion based on current guidelines [3]. Following a complete history, the examination will include PGP pain provocation tests and functional tests, in particular, but not exclusively, the posterior pelvic pain provocation (P4) test and the functional active straight leg raise (ASLR) test [19–22], which have been shown to have high sensitivity and specificity. In addition, other body areas that might be the source of a patient complaint will be examined to rule out other diagnoses. Participants must also be able to fluently speak and understand the local language (English, Spanish, or Swedish). Interviews will be transcribed and analysed using thematic analysis.

#### Methodological study 1: A systematic review with or without patient interviews to identify potential outcomes

When examining existing COS development studies, some authors include patient interviews in establishing the initial list of potential outcomes but others only search the literature [23]. Current guidance recommends that potential relevant outcomes are identified from several sources: systematic reviews of published studies, reviews of qualitative work, examination of items collected in national audit data sets, and interviews/focus groups with key stakeholders [14]. However, no studies have examined the impact of including patient interviews compared to conducting a systematic review only to identify the initial list of potential outcomes, on the final COS. Including interviews with participants presents additional costs and workload to the COS development team, including additional ethics applications, time and resources. It may be that the inclusion of patient representatives in the Delphi survey and in the consensus meeting makes patient interviews at the outcome identification stage redundant, but this is unknown. We will assess how the outcomes that are derived from the interviews only (if any) are rated in each round of the survey, and how many/type (if any) are subsequently included in the final PGP-COS. We will also examine the extent to which additional outcomes provided by patient representatives in round 1 of the Delphi survey overlap with the outcomes obtained from the interviews.

### Phase two: Online international Delphi survey

A sequential 3-round electronic, international Delphi study will be conducted including key stakeholders (patients/public, healthcare professionals, researchers, and service providers) to produce a preliminary PGP-COS. We will use online SurveyMonkey software to conduct the survey (www.surveymonkey.com). Each round will remain open for 14 days and a reminder email will be sent out four working days before closure. The data from each round will be analysed and presented to participants in the subsequent round. We will assess attrition rates for each round for all stakeholder groups.

#### Participants

We will invite people from all stakeholder groups, aiming for approximately 40 experts from each stakeholder group (patients, clinicians, researchers, service providers/policy makers) to participate (which will provide 20 people randomised to each scale; see methodological study 2). We will recruit participants for the Delphi survey through professional organisations, electronic discussion lists, and patient organisations. We will also encourage snowball sampling, asking participants to forward the invitation appropriately.

Eligible participants will be: (a) Researchers who are actively involved in research related to PGP/lumbopelvic pain and have at least one peer-reviewed publication on the topic; (b) Clinicians, currently treating patients with PGP (including chiropractors, physiotherapists, osteopaths, orthopaedic surgeons etc.); (c) Patients with PGP; (d) Policy-makers, involved in formulating policies related to the management of musculoskeletal conditions including PGP; and (e) Service providers of services for musculoskeletal conditions including PGP. Participants must be able to read and understand English in order to participate.

#### Round 1

Round 1 will collect demographic data including nationality, age, gender, stakeholder group and profession. Participants will rate the importance of each listed outcome. Outcomes will be rated in each round on a 9-point scale [16] or on a 5-point scale (see methodological study 2 below). Participants will also be given the opportunity to add any additional outcomes not already in the list that they think are important. All outcomes from round 1 will be forwarded to round 2.

#### Round 2

Participants who responded to round 1 will be presented with feedback (descriptive statistics) on their own and others’ scores from round 1 and asked to rescore all outcomes and score new outcomes added in round 1. The reason for providing feedback from the previous round to participants is to increase the degree of consensus [14]. Participants will rate outcomes using the same scale they used in round 1. Outcomes from round 2 will be forwarded to round 3 where 70% or more of participants score the outcome as ‘important’ (7 or more on the 9-point scale, or 4 or more on the 5-point scale), and less than 15% of participants scoring an outcome as ‘not important’ (3 or less on the 9-point scale, or 2 or less on the 5-point scale) (Tables 2 and 3).

**Table 2:** The 9-point rating scale

	9-point scale								
Outcome	Not important			Unsure of importance			Important		
Pain	1	2	3	4	5	6	7	8	9
	○	○	○	○	○	○	○	○	○

**Table 3:** The 5-point rating scale

	5-point scale				
Outcome	Not important		Unsure of importance	Important	
Pain	1	2	3	4	5
	○	○	○	○	○

#### Round 3

Round 3 will further prioritise outcomes by rescoring the outcomes based on round 2 feedback. Only participants who completed round 2 will be invited to round 3. Outcomes scored by 70% or more of participants as ‘important’ (7 or more on the 9-point scale, or as 4 or more on the 5-point scale), and less than 15% of participants scoring an outcome as ‘not important’ (3 or less on the 9-point scale, or 2 or less on the 5-point scale) will be included in the preliminary PGP-COS. At the end of the round 3 survey we will also include a question to ask if they would be willing to take part in the face-to-face consensus meeting.

#### Methodological study 2: Different scales to rate outcome importance

The 9-point scale, whereby a score of 1–3 means limited importance, 4–6 means maybe important but not critical, and 7–9 means critically important, is commonly used in Delphi surveys for COS development [14] and is based on the recommendations of the Grading of Recommendations Assessment, Development and Evaluation (GRADE) working group to assess the importance of evidence. However, other scales have been used [24, 25], and comments from some participants in round 1 of an in-progress recent Delphi survey described the 9-points scale as somewhat confusing and that a smaller scale might have been more user friendly [26]. Currently, no studies have examined the impact of using different likert scales on the final COS. We aim to compare the use of a 9-point scale (Table 2) versus a 5-point scale (Table 3). Participants of the Delphi survey, when clicking on the link to the survey, will be randomised to completing the survey using either a 9-point or 5-point scale. Specific software has been developed to enable this [27]. We will compare the two groups, with regards to the following outcomes: (a) the proportion of outcomes rated as ‘important’ (7 or above on 9-point scale, and 4 or 5 on 5-point scale) in each round and any differences between the scales, in the outcomes that are included in the preliminary COS after round 3. We will use z-scores to test the differences in proportions between the groups, and calculate overall mean scale differences on each outcome to assess differences. Recognising that differences can occur due to chance, and allowing for a 20% possibility of this, we would anticipate balance between the groups due to random allocation and thus similarity in overall scale scores for each outcome by population group. We will also ask participants to rate the ‘ease of use’ of the rating scale to assess utility.

### Phase three: Identification of outcome measurement tools

#### Conceptual considerations

The constructs (outcomes) to be measured will be identified in phase 2 and the target population has been described in the scope. Initially, we will conduct a systematic search of the literature to identify systematic reviews of outcome measurement instruments for the outcomes included in the preliminary PGP-COS derived from phase 2. We will search PubMed, Embase and the COSMIN database from inception. If a high quality systematic review has been conducted for the measurement of a particular outcome, including a quality assessment of the outcome measurement instruments, we will use this. If no high quality systematic review is identified for a particular outcome, then we will conduct a systematic review of outcome measurement instruments for that outcome, according to the COSMIN guidelines [15]. The systematic review(s) will be registered with PROSPERO (International Prospective Register of Systematic Reviews).

#### Search strategy & selection

We will search PubMed, Embase, and PEDro databases from inception for studies of outcome measurement instruments. The search strategy will be based on the search filter for finding measurement properties of measurement instruments, developed by Terwee et al. [28]. In addition, we will screen reference lists and contact experts. Eligibility of studies will be determine by two independent reviewers and in case of disagreement, consensus will be reached through discussion.

#### Quality assessment

Quality assessment will be conducted using the COSMIN checklist [29]. The feasibility of use in clinical practice and in research of the outcome measurement instrument(s) will also be assessed and further discussed in the consensus meeting (phase 4).

#### Recommendations

We will make recommendations on selecting outcome measurement instruments for the preliminary PGP-COS based on the quality of evidence and feasibility of outcome measurement instruments, which will be put forward to the consensus meeting (phase 4).

### Phase four: Consensus meeting

A face-to-face consensus meeting with a purposively sampled international panel representing views of all key stakeholders will be held to discuss, vote and agree on the final PGP-COS and associated methods (instruments) for measuring the outcomes. We will aim to select only one outcome measurement instrument per outcome where appropriate [15]. The meeting will include a presentation of the preliminary PGP-COS and outcome measurement instruments, followed by a timed discussion and voting. Consensus will be defined as at least 70% of participants voting for inclusion of an outcome/outcome measurement [30, 31] and at least one patient representative voting for inclusion of the outcome in the final COS.

#### Participants

A purposive sample of approximately 20 experts will form the consensus panel. The following stakeholder groups will be included: (a) Researchers who are actively involved in research related to PGP and have at least one peer-reviewed publication on the topic*;* (b) Clinicians currently treating patients with PGP (including chiropractors, physiotherapists, osteopaths, orthopaedic surgeons etc.); (c) Women (pregnant/postpartum) with PGP; (d) Policy-makers and service providers involved in formulating policies/providing services related to the management of musculoskeletal conditions, including PGP. The meeting will be held in English and translators may be required.

### Phase five: Dissemination and implementation strategy

A multi-method approach to dissemination will be adopted. The development of this PGP-COS will be reported according to the COS-STAR (Core Outcome Set-STAndards for Reporting) guidelines [32]. The final COS will be published in an open access journal and will be accompanied by a brief explanatory document with examples of good reporting to facilitate the use of the COS in practice and research. We will adhere to the authorship guidelines of the International Committee of Medical Journal Editors. Lay summaries will be included in dissemination documents. After publication, it will also be made available through the COMET database and we will register the COS with COMET and with CROWN (Core Outcomes in Women’s & Newborn Health). The systematic reviews of outcome measurement instruments will be submitted to the COSMIN database of systematic reviews of measurement properties.

In addition, we plan to disseminate the COS at national and international conferences and through relevant professional and patient organisations to inform healthcare professionals and the public. A summary of the COS will be translated in multiple languages. We will share the COS with clinical trial registries, consumer groups of relevance (e.g. maternity service user groups) and specific interested groups (e.g. World Federation of Chiropractic, World Confederation of Physical Therapy, North American Spine Society etc.) and we will ask all collaborators and Delphi panel participants to do this in their respective countries and promote snowball dissemination. Moreover, we will send the COS to international guideline development groups, policy makers, journal editors and funders of research in the area of PGP.

### Sample size

In phase 1, for the qualitative interviews, a purposive sample of 15 patients will be interviewed (5 in Ireland, 5 in Mexico, and 5 in Sweden). There is no consensus on methods for determining the sample size in qualitative research. The concept of data saturation is sometimes used, which is defined as the point when no new codes arise; however, this has been contested because the decision to cease recruitment remains subjective [33]. Subsequently, we based the sample size on previous COS development projects and guidance [16, 34].

For phase 2, limited guidance is available regarding methodological considerations using the Delphi technique [18] and sample size is not based on power calculation [14]. Subsequently, we based our sample size on existing COS development projects in the field [35]. Response rates in Delphi studies for COS can be low [29], hence we will invite sufficient people from all stakeholder groups, aiming for approximately 40 experts from each stakeholder group (patients, clinicians, researchers, service providers/policy makers) to participate (which will provide 20 people randomised to each scale; see methodological study 2).

In phase 5, a purposive sample of 20 experts will form the consensus panel. The COMET handbook does not specify a recommended number of participants to attend the consensus meeting [14] and sample size for this project has been determined to ensure that it is large enough to include multiple stakeholders (at least two for each stakeholder group) from different countries, ensuring adequate international representation, while minimizing costs.

### Ethical approval

University ethics approval has been obtained from Trinity College Dublin, the University of Dublin (School of Nursing & Midwifery Research Ethics Committee). For the qualitative interviews, patients will receive verbal and written information and will give informed consent prior to the interview. All transcripts will be confidential and will be given a number. Only the respective team interviewing the participants (in that specific country) will have access to the personal details of interviewees. They will have the right to discontinue the interview any time. Participants of the Delphi survey will receive all information regarding the study as part of the invitation email. Once they choose to participate, at the beginning of round one of the e-survey, they will consent to take part in the study. All personal data of participants will only be accessible to members of the research team and any response to the survey will be confidential. Participants will have the right to withdraw at any point. Model consent forms are provided in Additional file 1.

## Discussion

Currently, no published COS exists for PGP. It is essential that outcomes that are measured to assess and monitor a patient’s condition in research and in clinical practice are relevant to the patient. A well-developed and fully disseminated PGP-COS will ensure that relevant outcomes are measured using appropriate measurement instruments for women with PGP globally. Patients’ participation throughout this project will guarantee their input. This standardised COS will encourage effective monitoring, increase trial efficiency, improve evidence synthesis and reduce research waste to speed up the development and testing of treatment and prevention strategies. One of the challenges to this will be effective uptake of the COS by researchers. Insufficiency of uptake in COS was highlighted recently in a review of outcome domains for neuropathic pain [36]. In this study, outcomes from systematic reviews of randomised trials were reviewed and compared with the recommended IMMPACT (Initiative on Methods, Measurement, and Pain Assessment in Clinical Trials) COS. The study found 240 different outcome measures reported across 97 systematic reviews indicating insufficient use of relevant recommended COS in this condition. In moving forward with COS uptake a more concerted effort towards implementing COS is required.

## Additional file

Additional file 1: Model consent forms. Provides a copy of the consent forms to be used for the interviews (phase 1) and the Delphi survey (phase 2). (PDF 108 kb)

## Abbreviations

COMET: Core Outcome Measures for Effectiveness Trials
COS: Core Outcome Set
COSMIN: Consensus-based Standards for the selection of health Measurement INstruments
PGP: Pelvic Girdle Pain
