# Supplementary material for: Protocol for the development of a core outcome set for pelvic girdle pain, including methods for measuring the outcomes: the PGP-COS study
Source: BMC Med Res Methodol. 2018 Dec 3;18:158. doi: 10.1186/s12874-018-0624-5 (PMC6276248; doi:10.1186/s12874-018-0624-5)
Supplement: Supplementary file 1 — Model consent forms. Provides a copy of the consent forms to be used for the interviews (phase 1) and the Delphi survey (phase 2). (PDF 108 kb) [file 12874_2018_624_MOESM1_ESM.pdf]

Additional file 1: Model Consent forms

Trinity College Dublin
The University of Dublin

CONSENT FORM for interview

Research title: Development of a core outcome set for Pelvic Girdle Pain

Researcher: **Francesca Wuytack Tel: 087 51299776**

DECLARATION by participant: Please tick (X or √) and provide your initials

**1.**	I have read the information leaflet for this research study and I understand the contents.	**Yes [ ] No [ ] initials [ ]**
**2.**	I have had the opportunity to ask questions and all my questions have been answered to my satisfaction.	**Yes [ ] No [ ] initials [ ]**
**3.**	I fully understand that my participation is completely voluntary and that I am free to withdraw from the study and this interview at any time (prior to the data being merged for analysis) without giving a reason and that this will not affect my care in any way.	**Yes [ ] No [ ] initials [ ]**
**4.**	I understand that the transcript will not identify me by name but will use the study code and that the original digital recording will be erased once the accuracy of the transcript has been confirmed.	**Yes [ ] No [ ] initials [ ]**
**5.**	I understand that I will be given an opportunity to review the transcript from this interview to confirm accuracy.	**Yes [ ] No [ ] initials [ ]**
**6.**	I understand that the information from this research and this interview will be published but that I will not be identified as a participant in this research in any publication.	**Yes [ ] No [ ] initials [ ]**
**7.**	I agree that information obtained from me in this research and this interview which has been coded so as not to identify me may be stored and used for the purpose of future research which will have obtained research Ethics Committee approval without the need for further consent from myself.	**Yes [ ] No [ ] initials [ ]**

**8.**	I understand that my personal details (name and address and other identifying information that links me to the study data) will be destroyed when this study is complete unless I have agreed to its retention after that date and to being contacted about future research.	**Yes [ ] No [ ] initials [ ]**
**9.**	I freely and voluntarily consent to participating in this interview.	**Yes [ ] No [ ] initials [ ]**

PARTICIPANT'S NAME:

……………………………………………………………………………………………………..

Contact Address:

……………………………………………………………………………………………………..

……………………………………………………………………………………………………..

Phone number: ………………………………………………….. **Email:** ……………………….

Participant's signature: ……………………………………… **Date:**………………………..…..

Researcher: ……………………….. **Signature:** ………………………... Date:……………..……

One copy of this form must be retained by the participant and one copy must be retained by the researcher

Delphi survey: Consent form

Consent from participants

When accessing the Delphi survey, participants will be asked the following question:

"I consent to participate in this 3-round Delphi survey and agree to be contacted for all rounds of this Delphi survey." (yes/no)

"I consent to being contacted with an invitation to participate in a face-to-face consensus meeting which will be held following this Delphi survey." (yes/no)
